# The Narrowing Self: Depressive Tendency Attenuates the Mother‐Reference Effect

**DOI:** 10.1155/da/2882097

**Published:** 2026-08-26

**Authors:** Mingliang Gong, Shuanghong Chen

**Affiliations:** ^1^ School of Psychology, Jiangxi Normal University, Nanchang, China, jxnu.edu.cn

**Keywords:** depressive tendencies, mother-reference effect, self-concept, self-reference effect

## Abstract

In East Asia, close others (e.g., mothers) are often integrated into the self‐concept. However, individuals with high depressive tendencies (HDTs) exhibit a self‐boundary that is less inclusive of close others. The present study investigated whether HDT individuals in East Asian cultures include their mothers in their self‐concept. Experiment 1 revealed that low depressive tendency (LDT) participants showed a higher recognition accuracy for items transiently associated with mother compared to items transiently associated with others, indicating a mother‐reference effect (MRE), whereas HDT participants did not show this effect. Experiment 2 used the recollection/familiarity (R/K) paradigm to distinguish memory processing depth. The results revealed that LDT exhibited an MRE comparable to the self‐reference effect (SRE), whereas HDT participants showed a weaker MRE. HDT participants showed an MRE only in K responses, suggesting that their memory advantage for mother‐related information relies more on shallow processing. Experiment 3 employed the event‐related potential (ERP) technique with a shape‐label matching task, in which participants judged whether a geometric shape matched a subsequently presented identity label (self, mother, or stranger). The results showed that LDT participants demonstrated a MRE in both the N1 (70–120 ms) and N2 (220–280 ms) component amplitudes. In contrast, HDT participants showed this advantage only in the N2 component, suggesting delayed and shorter‐lasting MRE. Together, these findings demonstrate that depressive tendencies impair the MRE, reflecting reduced integration of mothers into the self.

## 1. Introduction

Humans, distinct from other animals, possess unique self‐awareness, rendering them more sensitive to stimuli related to themselves. A classic example is the cocktail party effect [1], where one can selectively recognize their own names amid noise. Empirical studies have further documented the self‐reference effect (SRE) [2], whereby self‐related information is remembered better than other‐related information. SRE manifests not only in long‐term self‐associated stimuli, such as one’s own face [3, 4], name [5, 6], and personal belongings [7], but also in brief self‐associated stimuli [8, 9]. For instance, Kim and Johnson [9] used an ownership paradigm where items were randomly assigned to baskets labeled “mine” or “Alex.” Memory tests showed better recognition for briefly self‐owned items than for others’ items.

Interestingly, this cognitive advantage may extend beyond the individual level to include close others (e.g., [10]), thereby forming an extended self [11]. Among these close others, mothers hold a unique position as individuals’ primary attachment figures [12]. Extensive research has demonstrated that mother‐related stimuli exhibit a significant cognitive processing advantage over stranger‐related stimuli—a phenomenon termed the mother‐reference effect (MRE) [13, 14]. Notably, this effect is particularly prominent in East Asian cultural contexts [10, 14–16], likely due to the emphasis on collectivist values in these cultures (e.g., [17]). In contrast, individuals from Western individualistic cultures typically show a SRE that is significantly stronger than the MRE, with the latter often failing to reach significance [16, 18, 19], suggesting that the inclusion of close others in the self‐concept is modulated by the cultural context. Collectivist values foster interdependent self‐concepts and cognitive structures [19, 20]. Neuroimaging studies have shown similar activation in the medial prefrontal cortex (MPFC) and anterior cingulate cortex (ACC) for self and mother [19, 21], suggesting a shared neural substrate for representing self and mother [22]. Thus, the MRE indicates that the self‐concept includes mothers [10, 14, 23–25].

However, the inclusion of mothers in the self‐concept may vary with individual traits. Beck’s cognitive theory of depression [26, 27] posits that depressed individuals exhibit a “depressive triad”—negative views of themselves, the world, and the future. They show reduced attention to the external environment, reflecting a narrower scope of the self that focuses on the “core self” [28]. As a result, the extended self may be stripped away from the self‐concept. Individuals with high depressive tendency (HDT) score high on depression scales but do not meet the criteria for clinical diagnosis. They differ from clinically depressed patients in symptom severity, degree of functional impairment, and patterns of emotional reactivity [29, 30]. Nevertheless, depressive symptoms in these two populations exist on a continuum rather than as discrete categories [31]. In many other aspects, HDT individuals demonstrate similar patterns to those with clinical depression (e.g., [32]). Using an oddball task in which participants viewed self‐relevant names (own, father’s, Chinese celebrity, and foreign celebrity), a standard stimulus and a target stimulus and only pressed a button for the target, Zhong et al. [32] found that individuals with low depressive tendencies (LDTs) showed larger P3 amplitudes in response to both their own names and their fathers’ names compared to nonself‐related names (i.e., foreign celebrities’ names). In contrast, HDT individuals only showed larger P3 amplitudes to their own name, with no significant distinction between fathers’ names and nonself stimuli. This indicates that LDT individuals exhibit both SRE and the father‐reference effect, whereas HDT individuals only retain SRE. Therefore, depressive tendencies may narrow self‐related processing to highly personalized stimuli, reflecting a simplified self‐concept. This narrowing of self‐related processing may extend beyond family members to other close social ties. Kokici et al. [33] reported that heightened depression (and loneliness) was linked to an exaggerated SRE alongside a diminished reference effect for close others, reflecting an increased cognitive distance between the self and close others. Together, these findings suggest that depressive tendencies and related affective states may systematically reduce the inclusion of various close others in the self.

In summary, previous research indicates that mothers are included in the concept of self, especially in East Asian cultures (e.g., [10]). However, HDT individuals focus on the personal core self [28], at least excluding the extended self of fathers [32]. This raises a critical question: Do the self‐concepts of HDT individuals in East Asian cultures still include the extended self of the mother? To answer this, the present study used two behavioral studies and one event‐related potential (ERP) study to investigate whether MRE is present in Chinese HDT individuals. Experiment 1 adopted the ownership paradigm to assess whether depressive tendencies modulate MRE for briefly associated stimuli. Experiment 2 used the R/K paradigm to examine the MRE for long‐term associations in HDT individuals. We hypothesized that LDT individuals would exhibit SRE and MRE for both stimuli briefly associated with the self and those with long‐term associations with the self, while HDT individuals would show only SRE, with a weak or absent MRE. Experiment 3 utilized ERP to compare the time course of mother‐reference processing between the HDT and LDT groups, focusing on N1, N2, and P3 components. N1 represents early perceptual processing [34] and reflects the allocation of attention to stimuli before stimulus evaluation [35]. N2 reflects attention engagement and stimulus categorization during visual stimulus processing [36–38] and is linked to self‐relevance [39]. P3 is an ERP component sensitive to self‐relevance, with larger amplitudes typically observed for self‐related stimuli than for other‐related stimuli [40, 41]. Additionally, a larger P3 amplitude is thought to reflect greater allocation of cognitive resources (e.g., [42]). We hypothesized that LDT individuals would show significantly larger N1, N2, and P3 amplitudes for self and mother versus other conditions, while these advantages would be weakened or absent in HDT individuals.

## 2. Methods

### 2.1. Experiment 1

#### 2.1.1. Participants

According to G ^∗^power 3.1.9 [43], the minimum required sample size for this experiment was 46 (*f* = 0.25, *α* = 0.05, 1‐*β* = 0.9). Participants were screened in two steps: First, 603 university students completed the Center for Epidemiologic Studies Depression Scale (CES‐D). Those scoring below 16 were categorized as LDT individuals (*n* = 35), while those scoring above 20 were classified as HDT individuals (*n* = 36). The cutoff score of 16 was originally proposed by Radloff [44] based on the 80th percentile in large community samples. A subsequent systematic review and meta‐analysis confirmed that this cutoff provides good screening accuracy for depression in epidemiological and primary care settings [45]. This standard has been widely adopted in research (e.g., Lin et al. [46]). After the experiment, participants completed the self‐rating depression scale (SDS), with index scores below 0.5 indicating LDT and above 0.5 indicating HDT [47]. Only participants consistently classified by both scales were included, which excluded 6 participants. After additionally excluding one participant due to key‐pressing errors, 64 participants were retained, including 32 LDT individuals (6 men; mean age = 22.16, SD = 1.92) and 32 HDT individuals (10 men; mean age = 20.87, SD = 1.52).

CES‐D scores differed significantly between groups (HDT: *M* = 28.78, SD = 1.54; LDT: *M* = 7.75, *SD* = 0.60), *t*(62) = 12.69, *p* < 0.001, Cohen’s *d* = 17.99. They also showed a significant difference on SDS scores (HDT: *M* = 0.62, *SD* = 0.12; LDT: *M* = 0.38, *SD* = 0.11), *t*(62) = 14.15, *p* < 0.001, Cohen’s *d* = 2.08. All participants had normal or corrected‐to‐normal vision, were right‐handed, signed informed consent forms before the experiment, and received compensation. This study was approved by the Institutional Review Board.

#### 2.1.2. Stimuli

We used 108 supermarket item pictures from Kim and Johnson [9]. Of these, 72 were used in the item assignment phase: 36 belonged to “mother” and 36 to “others.” The remaining 36 pictures served as new stimuli in the recognition phase, where they were presented alongside the 72 “old” stimuli from the item assignment phase. All pictures were 10.5 cm × 7.8 cm in size.

#### 2.1.3. Design and Procedure

A 2 (participant type: HDT and LDT) × 2 (reference type: mother and others) mixed design was used, with participant type as a between‐subjects variable and reference type as a within‐subject variable. The dependent variables were reaction time (RT) and accuracy in the recognition phase. The experiment comprised two phases: item assignment and item recognition.

Item assignment phase: A fixation point was presented in the center of the screen for 1000–1500 ms, followed by an item picture presented at the top of the screen for 2000 ms, alongside two identical baskets presented below. The two baskets were, respectively, labeled as “Mother’s” and “Zhang San’s” (a name commonly used among Chinese people to denote someone else). Subsequently, a red or green frame appeared around the item for 5000 ms, indicating the item’s ownership—red for “mother” (press “F”) and green for “Zhang San” (press “J”). Color ownership and key assignments were counterbalanced among participants. Finally, a blank screen was presented for 1000 ms. A total of 72 item pictures appeared in this phase.

Recognition phase: The recognition phase began immediately after the assignment phase, with no delay or distraction task in between. On each trial, an item picture appeared centrally after a 1000–1500 ms fixation point. Participants needed to judge if it was “old” (from the assignment phase) or “new” (unseen) by pressing “F” or “J” (keys were counterbalanced). After the key response, the item picture disappeared, followed by a 1000 ms blank screen. This phase included 108 trials (72 old and 36 new) presented randomly.

### 2.2. Experiment 2

#### 2.2.1. Participants

According to G ^∗^power 3.1.9, the minimum required sample size for this experiment was 36 (*f* = 0.25, *α* = 0.05, 1‐*β* = 0.9). The method of selecting participants was the same as in Experiment 1 (CES‐D cutoff scores: LDT < 16 and HDT > 20; SDS cutoff scores: LDT < 0.5 and HDT > 0.5). A total of 60 participants were recruited, including 29 LDT participants (6 men; mean age = 20.17, SD = 1.34) and 31 HDT participants (6 men; mean age = 20.23, SD = 1.34). HDT (*M* = 32.24, SD = 7.14) and LDT (*M* = 6.93, SD = 2.90) individuals significantly differed in CES‐D scores, *t*(58) = 17.74, *p* < 0.001, Cohen’s *d* = 4.64. They also differed significantly on SDS scores (HDT: *M* = 0.64, SD = 0.11; LDT: *M* = 0.39, SD = 0.08), *t*(58) = 10.41, *p* < 0.001, Cohen’s *d* = 2.60. All participants had normal or corrected‐to‐normal vision, were right‐handed, signed informed consent forms before the experiment, and received compensation.

#### 2.2.2. Stimuli

Two hundred and forty‐six adjectives (123 positive and 123 negative) were selected from the Chinese Affective Words System [48]. Of these, 6 words were used for practice and 240 words for the formal experiment. Among the 240 words in the formal experiment, 120 served as old words and were evenly assigned to three reference conditions: self, mother, and others. The assignment of specific adjectives to reference conditions was counterbalanced across participants (i.e., each adjective appeared equally often in each reference condition across different participants). The remaining 120 words were also evenly assigned to three reference conditions and served as new words for the item recognition phase. One‐way repeated‐measures ANOVAs showed no significant difference in familiarity [*F*(5, 114) = 0.62, *p* = 0.688] or arousal [*F*(5, 114) = 0.54, *p* = 0.744] across groups.

#### 2.2.3. Design and Procedure

The experiment adopted a 2 (participant type: HDT and LDT) × 3 (reference type: self, mother, and others) mixed design. Using the R/K paradigm, it consisted of three phases: learning, filler, and recognition.

In the learning phase, the three reference conditions (self, mother, and others) were presented in a fully randomized and intermixed design. On each trial, a fixation point was presented for 500 ms, followed by a reference condition and a trait adjective (e.g., “own name ‐ beautiful”). Participants rated how well the word described the name on a 1–5 scale within 4000 ms. Finally, a blank screen was presented for 1000 ms. The filler phase required participants to judge the parity of numbers randomly chosen from 1 to 300 within 1000 ms to prevent rehearsal.

In recognition, after a 500‐ms fixation, 120 old and 120 new trait adjectives were presented. Participants judged within 2500 ms whether they had seen the word in the learning phase. If “yes,” they needed to further make an R response (“Remember” judgment based on detailed recollection, e.g., “I remember seeing this word with ‘mother’”) or K response (“Know” judgment based on familiarity, e.g., “I know this word appeared but cannot recall the specific context”), with no time limit for R/K responses. A 1000 ms blank screen preceded the next trial.

### 2.3. Experiment 3

#### 2.3.1. Participants

The method of selecting participants was the same as in Experiment 1 (CES‐D cutoff scores: LDT < 16 and HDT > 20; SDS cutoff scores: LDT < 0.5 and HDT > 0.5). A total of 64 participants took part in this study. The predetermined cutoff criterion required that each condition retain at least 40 valid trials (i.e., >50% of the 80 trials per condition). Two participants were excluded for failing to meet this criterion due to excessive EEG artifacts, and another 2 were excluded due to inconsistent pre‐ and postexperiment depression scores, yielding a final sample of 60 participants. Among them, 30 were in the LDT group (5 men; mean age = 20.70, SD = 2.39), and 30 were in the HDT group (8 men; mean age = 20.30, SD = 1.56). HDT (*M* = 32.13, SD = 6.91) and LDT (*M* = 7.60, SD = 3.06) individuals differed significantly on CES‐D scores, *t*(58) = 17.78, *p* < 0.001, Cohen’s *d* = 4.59, and on SDS scores (HDT: *M* = 0.63, SD = 0.10; LDT: *M* = 0.38, SD = 0.11), *t*(58) = 9.37, *p* < 0.001, Cohen’s *d* = 2.38. All participants had normal or corrected‐to‐normal vision, were right‐handed, signed informed consent forms before the experiment, and received compensation.

#### 2.3.2. Stimuli

Three simple geometric figures (triangle, circle, and square) were used as stimulus materials, along with three reference‐type labels: self, mother, and stranger. The geometric figures were 3.8° × 3.8° in visual angle.

#### 2.3.3. Design and Procedure

A 2 (participant type: HDT and LDT) × 3 (reference type: self, mother, and stranger) × 2 (matching type: matching and mismatching) mixed design was adopted. Participant type was a between‐subjects variable, and reference type and matching type were within‐subject variables. The dependent variables included accuracy, RT, and the amplitudes of ERP components N1, N2, and P3.

The experiment included two sessions: the training stage and the matching stage. In the training stage, participants performed a 1‐min training task to associate shape labels (triangle, circle, and square) with identity labels (self, mother, and stranger). For example, they might learn that “The triangle represents yourself, the square represents your mother, and the circle represents a stranger that you are unfamiliar with.” Shape‐label pairings were counterbalanced across participants.

The trials in the matching stage started with a 500 ms fixation cross, followed by a 100 ms geometric figure, and then a random 200–500 ms blank screen. Next, an identity label was presented for 100 ms, after which a response screen appeared. Participants needed to judge whether the identity label matched the previous shape label within 1300 ms. The experiment included 480 trials, with 6 conditions (self‐matching, self‐mismatching, mother‐matching, mother‐mismatching, stranger‐matching, and stranger‐mismatching), each with 80 trials.

In this shape‐label matching task, participants must rapidly compare a geometric shape with a subsequent identity label (self, mother, and stranger). This process involves early perceptual attention (indexed by N1), stimulus categorization and attentional engagement (indexed by N2), and later controlled cognitive effort (indexed by P3). By manipulating reference type, the task allows us to examine whether mother‐related stimuli capture attention and engage categorization as strongly as self‐related stimuli do and whether this pattern differs between LDT and HDT groups.

#### 2.3.4. Data Acquisition and Analysis

Participants were seated 60 cm from a 17‐inch screen (1360 × 768 resolution) in a quiet room, with stimuli presented via the PsychoPy. A 64‐channel active Ag/AgCl electrode system (Brain Products ACTi Champ) positioned on an elastic cap according to the International 10–20 system was used to record the EEG signal. Bipolar electrodes were used to record the vertical electrooculogram (VEOG) and horizontal electrooculogram (HEOG). Electrode impedances were below 10 KΩ during data collection, and the sampling rate was 500 Hz. The online bandpass filter was set to 0.01–70 Hz.

Data analysis was performed using the EEGLAB toolbox [49], including (1) rereference: converting the online reference FCZ to offline references (the average of the left and right mastoids TP9 and TP10); (2) bandpass filtering: the EEG data were bandpass filtered (0.1–30 Hz) using a zero‐phase finite impulse response (FIR) filter (Hamming window) implemented in EEGLAB; (3) artifact removal: independent component analysis (ICA) was performed using EEGLAB. Eye blink and muscle components were identified using ICLabel [50], and all candidate components were further verified by visual inspection based on topographies, time courses, and power spectra; (4) segmentation and baseline correction: the signal was segmented into a series of 1200‐ms‐long epochs, each starting 200 ms before the onset of the identity label; (5) baseline correction: all epochs were baseline‐corrected to the mean of the 200 ms prestimulus period; (6) removing artifacts exceeding ± 100 μV; and (7) averaging trials under each condition for each participant.

Based on previous studies [51, 52] and visual inspection of grand averages, the electrodes and time windows for each component (N1, N2, and P3) were selected as follows: N1 was analyzed with a time window of 70–120 ms, using the average amplitude of FZ, F3, F4, CZ, C3, and C4; N2 was analyzed with a time window of 220–280 ms, using the average amplitude of CZ, C3, C4, CPZ, CP3, and CP4; and P3 was analyzed with a time window of 300–400 ms, using the average amplitude of CZ, C3, C4, CPZ, CP3, CP4, PZ, P3, and P4.

## 3. Results and Discussion

### 3.1. Experiment 1

#### 3.1.1. Results

**Figure 1:**
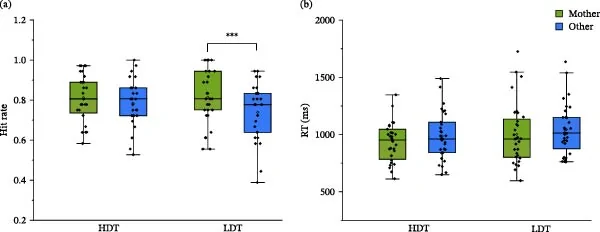
(a) Hit rates and (b) reaction times in Experiment 1. The error bars represent standard error of the mean. HDT: high depressive tendency; LDT: low depressive tendency.  ^∗∗∗^ *p* < 0.001.

A 2 (participant type: HDT and LDT) × 2 (reference type: mother and others) mixed ANOVA was conducted on the hit rate (i.e., the proportion of correct responses for old item trials). Response time was also analyzed as a complementary measure of processing efficiency and to assess the speed‐accuracy tradeoff. The results are shown in Figure 1.

For hit rate, the interaction was significant, *F*(1, 62) = 5.89, *p* = 0.018, $\eta_{p}^{2}$ = 0.087. Simple effects analysis showed that LDT participants had a significant hit rate for mother‐owned items (*M* = 0.80, SD = 0.17) than for other‐owned items (*M* = 0.72, SD = 0.18), *p* < 0.001, Cohen’s *d* = 0.87, 95% CI = [0.05, 0.11], while HDT participants showed no significant difference in hit rate between mother‐owned (*M* = 0.81, SD = 0.11) and other‐owned items (*M* = 0.79, SD = 0.11), *p* = 0.108, Cohen’s *d* = 0.30, 95% CI = [−0.01, 0.06]. The main effect of reference type was significant, *F*(1, 62) = 22.43, *p* < 0.001, $\eta_{p}^{2}$ = 0.266, while the main effect of participant type was not significant, *F*(1, 62) = 1.41, *p* = 0.239, $\eta_{p}^{2}$ = 0.022.

To assess potential response bias between groups, overall false alarm rates (i.e., proportion of “old” responses to new items) were compared. An independent samples *t*‐test showed that there was no significant difference in false alarm rates between HDT (*M* = 0.12, SD = 0.13) and LDT (*M* = 0.16, SD = 0.20) participants, *t*(62) = −1.01, *p* = 0.318, indicating that the two groups did not differ in their overall tendency to respond “old” to new items. Thus, the observed hit rate patterns are not confounded by the differential response bias.

For RT, the interaction was not significant, *F*(1, 62) = 1.02, *p* = 0.316, $\eta_{p}^{2}$ = 0.016. The main effect of reference type was significant, *F*(1, 62) = 4.92, *p* = 0.030, $\eta_{p}^{2}$ = 0.073, with significantly faster recognition of items belonging to the mother (*M* = 968.97 ms, SD = 222.52 ms) than those belonging to others (*M* = 1051.03 ms, SD = 354.85 ms), *p* = 0.030, Cohen’s *d* = −0.28, 95% CI = [−156.03, −8.09]. The main effect of participant type was not significant, *F*(1, 62) = 0.13, *p* = 0.725, $\eta_{p}^{2}$ = 0.002.

#### 3.1.2. Discussion

Experiment 1 used an ownership paradigm to explore whether HDT individuals show a recognition advantage for stimuli briefly associated with the mother. The results showed that LDT participants had a significantly higher hit rate for mother‐owned items compared to other‐owned items, demonstrating the MRE. In contrast, HDT participants showed no such difference, suggesting the absence of the MRE. This finding supports our hypothesis and aligns with Zhong et al.’s [32] findings on the father‐reference effect. However, the absence of MRE in HDT participants may be due to the fact that the hit rate alone is a relatively coarse measure, which may not be sufficiently sensitive to capture more subtle forms of mother‐related memory advantage. The R/K paradigm offers greater sensitivity to self‐reference processing by dissociating R‐based from K‐based responses [53, 54], providing a more fine‐grained assessment of memory. Therefore, Experiment 2 adopted this paradigm to further examine how depressive tendencies influence the MRE. Additionally, a self‐reference condition was included to directly compare the MRE and SRE.

### 3.2. Experiment 2

#### 3.2.1. Results

**Figure 2:**
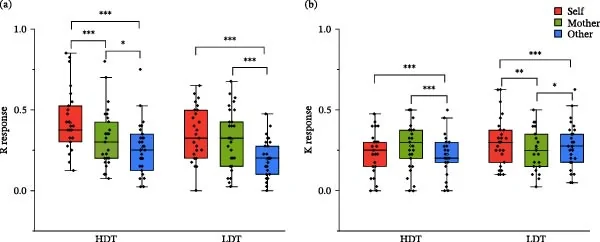
(a) The R responses and (b) K responses in Experiment 2. The error bars represent one standard error.  ^∗^ *p* < 0.05,  ^∗∗^ *p* < 0.01,  ^∗∗∗^ *p* < 0.001.

Hit rate, R response, and K response were calculated, and mixed ANOVAs were conducted on them (see Figure 2).

##### 3.2.1.1. Hit Rate

The interaction between participant type and reference type was not significant, *F*(2, 116) = 0.14, *p* = 0.868, $\eta_{p}^{2}$ = 0.002. The main effect of reference type was significant, *F*(2, 116) = 72.51, *p* < 0.001, $\eta_{p}^{2}$ = 0.556. Multiple comparisons with Bonferroni correction showed that the hit rate for adjectives matched with one’s own name (*M* = 0.65, SD = 0.18) was significantly higher than that for mother‐matched (*M* = 0.59, SD = 0.20), *p* < 0.001, Cohen’s *d* = 0.58, 95% CI = [0.03, 0.09] and others‐matched adjectives (*M* = 0.47, SD = 0.18), *p* < 0.001, Cohen’s *d* = 1.47, 95% CI = [0.14, 0.21]. Additionally, mother‐matched adjectives had higher hit rates than others‐matched, *p* < 0.001. The main effect of participant type was not significant, *F*(1, 58) = 0.17, *p* = 0.684, $\eta_{p}^{2}$ = 0.003.

Similar to Experiment 1, overall false alarm rates (i.e., proportion of “old” responses to new items) were compared to assess the potential response bias between groups. An independent samples *t*‐test (with correction for unequal variances if needed) showed that there was no significant difference in false alarm rates between HDT (*M* = 0.24, SD = 0.11) and LDT (*M* = 0.30, SD = 0.15) participants, *t*(49.13) = −1.63, *p* = 0.110, indicating that the two groups did not differ in their overall tendency to respond “old” to new items. Thus, the observed hit rate patterns are not confounded by the differential response bias.

##### 3.2.1.2. R Response

The interaction was significant, *F*(2, 116) = 3.76, *p* = 0.026, $\eta_{p}^{2}$ = 0.061. Simple effect analysis showed that for HDT participants, R response accuracy for self‐matched adjectives (*M* = 0.42, SD = 0.18) was significantly higher than for mother‐matched adjectives (*M* = 0.32, SD = 0.17), *p* < 0.001, Cohen’s *d* = 1.31, 95% CI = [0.07, 0.12], and others‐matched adjectives (*M* = 0.26, SD = 0.16), *p* < 0.001, Cohen’s *d* = 1.38, 95% CI = [0.12, 0.21]. Mother‐matched adjectives was also significantly higher than that others‐matched, *p* = 0.003, Cohen’s *d* = 0.60, 95% CI = [0.02, 0.11]. For the LDT group, R response accuracy for self‐matched (*M* = 0.34, SD = 0.17) and mother‐matched adjectives (*M* = 0.32, SD = 0.18) was higher compared to others‐matched (*M* = 0.20, SD = 0.12), *p*s < 0.001, but there was no significant difference between self‐matched and mother‐matched, *p* = 0.087. The main effect of reference type was significant, *F*(2, 116) = 63.59, *p* < 0.001, $\eta_{p}^{2}$ = 0.523. The main effect of participant type was not significant, *F*(1, 58) = 1.45, *p* = 0.233, $\eta_{p}^{2}$ = 0.024.

##### 3.2.1.3. K Response

Following Yonelinas [54], raw K rates underestimate familiarity because they are conditional on failed R. Thus, K was corrected using the following formula: Familiarity = *P*(K)/[1 − *P*(R)].

A two‐factor mixed ANOVA revealed a significant interaction, *F*(2, 116) = 3.59, *p* = 0.031, $\eta_{p}^{2}$ = 0.058. Simple effect analysis showed that, for HDT participants, the K response accuracy for self‐matched adjectives (*M* = 0.40, SD = 0.20) and mother‐matched adjectives (*M* = 0.41, SD = 0.21) was significantly higher than for other‐matched (*M* = 0.28, SD = 0.15), *p*s < 0.001, whereas there was no significant difference between self‐matched and mother‐matched adjectives, *p* = 0.644. For LDT participants, K response accuracy was significantly higher for self‐matched adjectives (*M* = 0.48, SD = 0.23) than mother‐matched (*M* = 0.40, SD = 0.20) and others‐matched (*M* = 0.36, SD = 0.19), *p* = 0.006 and *p*  < 0.001, respectively. Mother‐matched adjectives was also higher than others‐matched adjectives, *p* = 0.043. The main effect of reference type was significant, *F*(2, 116) = 23.04, *p* < 0.001, $\eta_{p}^{2}$ = 0.284, whereas the main effect of participant type was not significant, *F*(1, 58) = 0.49, *p* = 0.485, $\eta_{p}^{2}$ = 0.008.

#### 3.2.2. Discussion

Experiment 2 used the R/K paradigm, which is more sensitive to self‐reference processing [53] to dissociate R‐based memory (R responses) from K‐based processing (K responses). For R response, the results showed that LDT individuals exhibited an MRE comparable to the SRE, whereas HDT individuals showed an MRE significantly smaller than the SRE. This supports the view that individuals with depressive tendencies have a stronger focus on the core self [28, 32]. For K responses, HDT individuals showed an MRE comparable to their SRE, whereas LDT individuals showed an MRE that was weaker than that of their SRE. Taken together with the R response results, these findings indicate that the MRE in LDT individuals is based on deep recollective processing (primarily reflected in R responses), whereas the MRE in HDT individuals is based primarily on shallow K processing (reflected in K responses). Thus, depressive tendencies do not eliminate the MRE but rather degrade it from “remembering” to “knowing.” Therefore, the processing of mother‐related stimuli in HDT individuals is shallower than that in LDT individuals.

To further explore the differences in mother‐reference processing between HDT and LDT individuals and identify the specific stages driving these differences, Experiment 3 employed the ERP technique. Autobiographical information (e.g., one’s own name) used in Experiment 2 might confound SREs with K effects [55, 56]—the enhanced processing of pre‐experimentally familiar stimuli that can produce a memory advantage independent of genuine self‐referential encoding. To eliminate this potential confound, Experiment 3 adopted a shape‐label matching paradigm in which geometric shapes were arbitrarily assigned to identity labels (self, mother, and stranger). This ensures that any observed advantage reflects true self‐ or MREs rather than preexisting K.

### 3.3. Experiment 3

#### 3.3.1. Behavioral Results

Following previous studies [55, 57], we analyzed matching and mismatching conditions separately. Since the SRE has been shown to manifest only in the matching condition [56], and no interaction effect between participant type and reference type was observed under the mismatching condition in the present study (see the Supporting Information), we focused on the results from the matching condition, in line with the approach of Chen et al. [51].

##### 3.3.1.1. Accuracy

For the matching condition, the interaction between participant type and reference type was not significant, *F*(2, 116) = 1.10, *p* = 0.336, $\eta_{p}^{2}$ = 0.019. The main effect of reference type was significant, *F*(2, 116) = 45.79, *p* < 0.001, $\eta_{p}^{2}$ = 0.441. Multiple comparisons with Bonferroni corrections showed that the accuracy under the self condition (*M* = 0.96, SD = 0.04) was significantly higher than that under the mother condition (*M* = 0.94, SD = 0.06), *p* = 0.042, Cohen’s *d* = 0.34, 95% CI = [0.00, 0.03], and was also higher than the stranger condition (*M* = 0.87, SD = 0.09), *p* < 0.001. The accuracy under the mother condition was also significantly higher than that under the stranger condition, *p* < 0.001. The main effect of participant type was not significant, *F*(1, 58) = 0.30, *p* = 0.588, $\eta_{p}^{2}$ = 0.005.

##### 3.3.1.2. RT

For the matching condition, the interaction was not significant, *F*(2, 116) = 0.77, *p* = 0.466, $\eta_{p}^{2}$ = 0.013. The main effect of reference type was significant, *F*(2, 116) = 57.65, *p* < 0.001, $\eta_{p}^{2}$ = 0.498. Multiple comparisons with Bonferroni corrections revealed faster RT for the self condition (*M* = 447.67 ms, SD = 99.30 ms) compared to the mother condition (*M* = 487.42 ms, SD = 108.76 ms), *p* = 0.001, Cohen’s *d* = ‐0.48, 95% CI = [−65.00, −14.00], and the stranger condition (*M* = 552.71 ms, SD = 119.50 ms), *p* < 0.001, Cohen’s *d* = −1.34, 95% CI = [−129.00, −81.00]. The RT in the mother condition was also significantly faster than the stranger condition, *p* < 0.001, Cohen’s *d* = ‐0.84, 95% CI = [−89.00, −41.00]. The main effect of participant type was not significant, *F*(1, 58) = 0.28, *p* = 0.598, $\eta_{p}^{2}$ = 0.005.

#### 3.3.2. EEG Results

**Figure 3:**
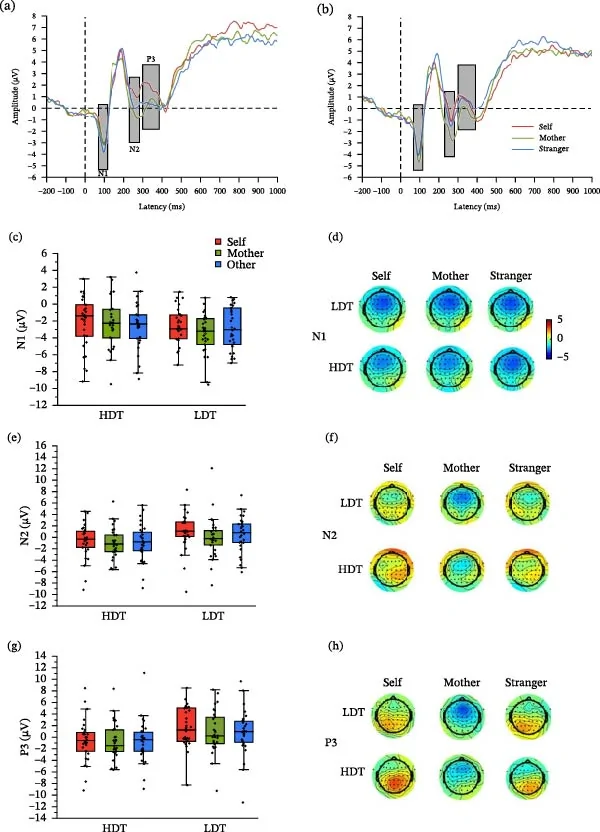
The ERP results in Experiment 3. (a, b) ERP waveshapes at a typical electrode Cz for HDT and LDT participants; (c, d) N1 amplitude averaged across electrodes in the N1 ROI and the topographic maps of N1 amplitude; (e, f) N2 amplitude averaged across electrodes in the N2 ROI and the topographic maps of N2 amplitude; (g, h) P3 amplitude averaged across electrodes in the P3 ROI and the topographic maps of P3 amplitude. Error bars represent standard error.

Since the self‐processing advantage is mainly reflected in the shape‐identity matching condition [51], only data under the matching condition were analyzed. The results are shown in Figure 3.

##### 3.3.2.1. N1 (70–120 ms)

The interaction between participant type and reference type was significant, *F*(2, 116) = 3.24, *p* = 0.043, $\eta_{p}^{2}$ = 0.053. Simple effect analysis showed that for HDT participants, N1 amplitudes under the self condition (*M* = −2.07 μV, SD = 3.08) was not significantly different from the mother condition (*M* = −2.12 μV, SD = 2.88) and the stranger condition (*M* = −2.57 μV, SD = 2.78), *p* = 0.876 and *p* = 0.138, respectively. The mother condition and the stranger condition also did not differ, *p* = 0.230. For LDT participants, N1 amplitudes under the mother condition (*M* = −3.49 μV, SD = 2.39) was larger than the self condition (*M* = −2.60 μV, SD = 2.09), *p* = 0.004, Cohen’s *d* = −0.55, 95% CI = [−1.48, −0.30], and marginally larger than the stranger condition (*M* = −2.77 μV, SD = 2.52), *p* = 0.060, Cohen’s *d* = −0.32, 95% CI = [−1.47, 0.03]. No significant difference was revealed between the self condition and the stranger condition, *p* = 0.613. The main effects of reference type and participant type were not significant, *F*(2, 116) = 2.05, *p* = 0.133, $\eta_{p}^{2}$ = 0.034 and *F*(1, 58) = 1.25, *p* = 0.268, $\eta_{p}^{2}$ = 0.021.

##### 3.3.2.2. N2 (220–280 ms)

The interaction was not significant, *F*(2, 116) = 0.31, *p* = 0.732, $\eta_{p}^{2}$ = 0.005. The main effect of reference type was significant, *F*(2, 116) = 13.52, *p* < 0.001, $\eta_{p}^{2}$ = 0.189. Multiple comparisons with Bonferroni corrections showed that the N2 amplitude induced under the mother condition (*M* = −0.46 μV, SD = 3.22) was significantly larger than the self condition (*M* = 0.96 μV, SD = 2.99), *p* < 0.001, Cohen’s *d* = −0.63, 95% CI = [−2.15, −0.70], and the stranger condition (*M* = 0.43 μV, SD = 3.02), *p* = 0.003, Cohen’s *d* = ‐0.45, 95% CI = [−1.53, −0.26]. However, no significant difference was found between the self and the other conditions, *p* = 0.189. The main effect of participant type was not significant, *F*(1, 58) = 1.68, *p* = 0.200, $\eta_{p}^{2}$ = 0.028.

##### 3.3.2.3. P3 (300–400 ms)

The interaction was not significant, *F*(2, 116) = 1.90, *p* = 0.154, $\eta_{p}^{2}$ = 0.032. The main effect of reference type was significant, *F*(2, 116) = 5.05, *p* = 0.008, $\eta_{p}^{2}$ = 0.080. Multiple comparisons with Bonferroni corrections showed that the P3 amplitude induced under the self condition (*M* = 1.66 μV, SD = 3.66) was significantly larger than the mother condition (*M* = 0.86 μV, SD = 3.66), *p* = 0.003, Cohen’s *d* = 0.45, 95% CI = [0.23, 1.37], but was not significantly different from the stranger condition (*M* = 1.27 μV, SD = 4.06), *p* = 0.422. The mother condition and the stranger condition did not differ, *p* = 0.366. The main effect of participant type was not significant, *F*(1, 58) = 0.28, *p* = 0.598, $\eta_{p}^{2}$ = 0.005.

#### 3.3.3. Discussion

Experiment 3 employed ERPs to examine temporal differences in mother‐referential processing between HDT and LDT individuals. Behaviorally, both groups showed similar patterns: performance in both mother‐reference and self‐reference conditions was superior to the stranger condition in terms of accuracy and RT, demonstrating both SRE and MRE. The behavioral MRE pattern observed in Experiments 1 and 2 was not replicated in Experiment 3, which can be attributable to paradigm differences. Experiments 1 and 2 used memory‐recognition tasks requiring encoding/retrieval of meaningful stimuli (adjectives and owned items), whereas Experiment 3 used a shape‐label perceptual‐matching task prioritizing rapid perceptual judgment over memory.

Nevertheless, the ERP data consistently revealed the core neural impairment—delayed MRE in HDT individuals—aligning with the main findings of the study. For N1 amplitudes, LDT individuals exhibited enhanced responses to mother‐referential stimuli compared to self and stranger conditions, while HDT individuals showed no such differentiation. For N2, both groups demonstrated significant MRE in N2 amplitudes, with no interaction between the reference type and participant type. P3 amplitudes revealed larger responses to self‐ than mother‐referential stimuli, though neither differed significantly from stranger conditions. Critically, while LDT individuals showed MRE effects in both early (N1) and mid‐latency (N2) components, HDT individuals only displayed MRE in N2. This suggests that mothers do not capture the attention of HDT individuals in the early stages of perceptual processing but do capture the attention of LDT individuals. These temporal differences in neural processing may underlie the weaker behavioral MRE observed in HDT individuals in Experiments 1 (absence of MRE in accuracy) and 2 (smaller MRE in R responses compared to LDT) compared to the robust MRE of LDT individuals.

## 4. General Discussion

The present study investigated how depressive tendencies influence the MRE in East Asian culture. Experiment 1 (ownership paradigm) revealed that items briefly associated with mothers were significantly better recognized than those associated with others in LDT participants, demonstrating a MRE. However, this MRE is absent in HDT participants. Experiment 2 (R/K paradigm) showed that LDT participants exhibited MRE comparable to SRE in the R response, while the MRE of HDT participants was weaker than that of LDT participants. Interestingly, only HDT individuals displayed MRE in K responses. Experiment 3 further revealed differences at the neural level: LDT participants showed larger N1 and N2 amplitudes for mother reference compared to stranger reference (i.e., MRE). In contrast, the HDT group only showed MRE in the N2 component. For P3, both groups showed larger amplitudes for self‐ versus mother‐reference conditions, with no significant MRE observed.

LDT individuals showed MRE under all three paradigms in the present study, and Experiment 2 showed that its strength was comparable to that of SRE. This indicates that LDT individuals have a strong and stable MRE. This aligns with prior research [10, 14–16], indicating that the self‐concept of East Asians includes mothers. Notably, Experiment 2 revealed that LDT individuals showed MRE in R responses but not in K responses, suggesting that their memory advantage for mother‐related stimuli arises from elaborative encoding rather than K. According to the self‐expansion model [11], individuals cognitively incorporate intimate others into their self‐concept to acquire new identities, perspectives, and resources. As a key role in the early socialization process, mothers play a crucial role in individuals’ emotional regulation, social development, and childhood attachment formation [58]. Within collectivist cultural contexts, LDT individuals stably incorporate their mothers into their self‐concept as an integral part [20].

Compared with LDT individuals, HDT individuals showed a less stable MRE. While Experiment 2 found MRE in R responses, its effect was smaller than that of SRE, and it completely disappeared in the brief ownership association task of Experiment 1. These findings indicate that HDT individuals’ MRE is more fragile than LDT individuals’ and align with previous research showing that HDT individuals are more focused on the core self [28, 32]. We also showed that, unlike the absence of K‐response MRE in LDT individuals, HDT individuals showed K‐response MRE, which was even larger than their SRE. This result suggests that HDT individuals rely more on a sense of K rather than specific details when recalling mother‐related information. Such shallow processing may impede the consolidation of mother‐related memories via elaborative encoding, leading to the instability of MRE.

Experiment 3 revealed distinct MRE patterns between groups, which may provide a neural‐level explanation for the behavioral results of Experiments 1 and 2. LDT individuals showed a processing advantage for mother‐related stimuli in the early (N1) and middle (N2) stages, which enables them to stably exhibit an MRE in the ownership task (Experiment 1) and detail R (as indexed by the R response in Experiment 2). In contrast, HDT individuals only showed a processing advantage for mother‐related stimuli in the middle (N2) stages but not in the early (N1) stage. This result suggests that the onset of MRE in HDT individuals is delayed compared to that in LDT individuals (i.e., MRE emerges later) or a qualitative deficit in early attentional allocation to mother‐related information. This may explain the unstable MRE observed in HDT individuals in the present study.

N1 reflects the automatic allocation of attentional resources in the perceptual stage [35], indicating that LDT individuals have an automatic attention bias towards mother‐related stimuli in the early stage of perceptual processing, whereas this is absent in HDT individuals. This may stem from the hyper‐focus of HDT individuals on their core self and their reduced attentional engagement with external stimuli [28], which prevents early attentional capture by mother‐related stimuli. Task demands—rapid shape presentation followed by identity judgments—likely amplify early attentional differences because successful performance depends on quick perceptual tagging. N2 reflects attentional engagement during visual stimulus processing [38], with its centro‐parietal subcomponent N2b reflecting the elaborate processing of stimulus classification and identification [36, 37]. This suggests that both LDT and HDT participants allocated more attention to mother‐related stimuli than to stranger‐related stimuli during the mid‐stage of processing, which in turn promoted more elaborate cognitive processing of such stimuli.

The ERP results also revealed significantly larger P3 amplitudes for the self compared to mother conditions. Since the P3 component is affected by attentional resources, with a larger amplitude indicating more investment of attentional resources [42], this result indicated that individuals devoted more attention to self‐related stimuli than to mother‐related stimuli in late processing. However, the present study did not observe enhanced P3 amplitudes for the mother condition relative to the stranger condition for both HDT and LDT participants, which is inconsistent with previous research [40, 41]. This discrepancy may stem from differences in experimental paradigms. McIvor et al. [55] employed autobiographical information, including self‐faces, and self‐names as stimuli, which risks conflating the SRE with the K effect [55, 56]. Fan et al. [40] used negative and neutral images as stimuli, where the inherent valence and arousal levels of the images may influence memory performance. In contrast, the current study adopted a shape‐label matching paradigm, in which geometric shapes were randomly assigned to “self,” “mother,” and “other” conditions. This design effectively avoids the confounding of self‐reference with K and eliminates the interference of stimulus valence and arousal with reference effects.

Our findings demonstrate that even within relationship‐oriented Eastern collectivist cultures, depressive tendencies weaken the stable MRE observed in LDT individuals. This reflects the interplay between cultural and clinical influences: collectivist culture fosters including mothers in self‐concepts (e.g., [14]), while depression drives pathological hyper‐focus on the “core self” (e.g., [28]). This informs culturally adapted intervention—in the practice of treating depression in the context of Eastern collectivist cultures, family‐focused approaches may work better than Western individual‐centered psychotherapies. Activating positive bonds between individuals and their mothers helps HDT individuals expand their self‐concept, thereby improving social cognitive dysfunction caused by self‐narrowing. However, caution is warranted when interpreting the clinical implications. First, in East Asian cultural contexts, mothers are already integrated into the self‐concept [19]; thus, for HDT individuals, the issue lies not in the absence of such integration but in its attenuation. Second, mother–child bonds in Asian families can also entail obligation and pressure rather than solely supportive self‐expansion [59]. Therefore, simply activating mother–child bonds may not automatically alleviate self‐narrowing. Instead, culturally adapted interventions for depression should focus on positive, autonomy‐supportive mother–child interactions—such as recalling warm memories or affirming mutual respect—rather than indiscriminately emphasizing filial bonds. This nuanced approach may help HDT individuals expand their self‐concept while avoiding potential distress from perceived obligation.

While the present study yields significant findings, several limitations warrant discussion. First, all three experiments featured a female‐dominated sample (~75%–80% female). This imbalance is noteworthy for two reasons. On the one hand, depressive tendencies are more prevalent in females than in males [60], and our sample composition broadly reflects this population trend. On the other hand, in East Asian cultural contexts, mother–child closeness and filial responsibility often differ by gender, with daughters typically reporting greater emotional intimacy with mothers than sons do [61]. Since closer mother–child relationships increase the likelihood of incorporating the mother into the self, the present findings may overestimate the MRE, as future studies with balanced gender samples are needed to determine whether the attenuation of MRE in HDT individuals generalizes across genders or interacts with participant gender. Alternatively, future research could take closeness as a variable to further explore its impact on self‐concept construction and MRE in individuals with and without depression tendencies. Second, while the R/K paradigm dissociates R from K, future research can adopt confidence rating measures [62] to explore the subjective metacognitive characteristics of mother‐reference processing in individuals with different depressive tendencies, which will provide a more comprehensive understanding of the memory processing of mother‐related information. Third, depressive tendencies differ from clinical depression [63], so the findings apply only to nonclinical HDT groups. Further research should test their generalizability to clinically depressed populations. Finally, the present study was conducted exclusively in an East Asian collectivist cultural context. Future research can replicate the present paradigm in Western individualistic samples to explore the cross‐cultural universality or specificity of the impairment of MRE by depressive tendencies. This will further reveal the interaction mechanism of cultural background, depressive traits, and close‐other reference processing.

In conclusion, LDT individuals exhibit a robust and stable MRE, demonstrating an early processing advantage for mother‐related stimuli. In contrast, while HDT individuals also exhibit an MRE, it is less robust compared to that of their LDT counterparts, characterized by shallower processing of mother‐related stimuli and a delayed emergence of the MRE in later processing stages. In other words, the MRE in HDT individuals is significantly weaker than that in LDT individuals, both behaviorally and neurologically. These findings not only demonstrate that depressive symptoms have a regulatory effect on culturally shaped self‐cognitive patterns but also provide a new perspective from cultural psychology for understanding the pathological mechanism of depression.

## Funding

This work was supported by the National Natural Science Foundation of China (Grant 32460204) and the Social Science Planning Project of Jiangxi Province (Grant 21JY47).

## Ethics Statement

The study was approved by the Review Board of the School of Psychology, Jiangxi Normal University (Number IRB‐JXNU‐PSY‐2021022).

## Conflicts of Interest

The authors declare no conflicts of interest.

## Supporting Information

Additional supporting information can be found online in the Supporting Information section.

## Supporting information


**Supporting Information** Supporting Information provides the complete behavioral results (accuracy and reaction time) for the mismatching condition of Experiment 3.

## Data Availability

The data for the experiments reported here are available on the Open Science Framework at https://osf.io/6xzn2/overview?view_only=8dc25b87dc5c443197b982f4cac1a881.
