# Supplementary material for: The Narrowing Self: Depressive Tendency Attenuates the Mother‐Reference Effect
Source: Depress Anxiety. 2026 Aug 26;2026:2882097. doi: 10.1155/da/2882097 (PMC13509424; doi:10.1155/da/2882097)
Supplement: Supplementary file 1 [file DA-2026-2882097-s001.docx]

**Supplementary material**

**Behavioral Results for the Mismatch Condition in Experiment 3**

**(1) Accuracy**

Mismatching condition: The interaction was not significant, *F*(2, 116) = 1.99, *p* = .142, *η2 p* = .033. The main effect of reference type was marginally significant, *F*(2, 116) = 3.03, *p* = .052, *η2 p* = .050. Multiple comparisons with Bonferroni corrections showed that the accuracy under the self condition (*M* = .93, *SD* = .07) was not significantly different from that under the mother condition (*M* = .93, *SD* = .07), *p* = .781, but higher than that under the stranger condition (*M* = .92, *SD* = .06), *p* = .031, Cohen’s *d* = .34, 95% CI = [.00, .03]. No significant difference was revealed between the mother condition and the stranger condition, *p* = .659. The main effect of participant type was not significant, *F*(1, 58) = .62, *p* = .434, *η2 p* = .011.

**(2) Reaction time**

Mismatching condition: The interaction was not significant, *F*(2, 116) = .09, *p* = .915, *η2 p* = .002. The main effect of reference type was significant, *F*(2, 116) = 5.43, *p* = .006, *η2 p* = .086. Multiple comparisons with Bonferroni corrections showed no significant RT difference between self (*M* = 547.53 ms, *SD* = 109.14 ms) and mother condition (*M* = 554.86 ms, *SD* = 114.24 ms), *p* = .865. However, the RT in the self condition was significantly faster than the stranger condition (*M* = 568.10 ms, *SD* = 118.99 ms), *p* = .011, Cohen’s *d* = -.38, 95% CI = [-37.00, -4.00]. The mother condition also showed faster RT compared to the stranger condition, *p* = .042, Cohen’s *d* = -.32, 95% CI = [-26.00, .00]. The main effect of participant type was not significant, *F*(1, 58) = .18, *p* = .672, *η**2 p*= .003.
